# Transurethral Unroofing of a Symptomatic Imperforate Cowper's Syringocele in an Adult Male

**DOI:** 10.1155/2016/3743607

**Published:** 2016-03-28

**Authors:** Mohannad A. Awad, Amjad Alwaal, Catherine R. Harris, Uwais B. Zaid, Thomas W. Gaither, E. Charles Osterberg, Benjamin N. Breyer

**Affiliations:** ^1^Department of Urology, University of California San Francisco, San Francisco, CA 94117, USA; ^2^Department of Urology, King Abdul Aziz University, Jeddah 21589, Saudi Arabia; ^3^Division of Urology, Duke University, Durham, NC 27710, USA

## Abstract

Cystic dilatation of bulbourethral gland ducts (Cowper's gland syringocele) is a rare abnormality. The condition has been described among pediatric populations, but it is uncommon in adults. It can be asymptomatic or present with obstructive and irritative urinary symptoms. We report a case of a symptomatic imperforate Cowper's syringocele in a young patient that was successfully managed with transurethral unroofing of the cyst.

## 1. Introduction

Bulbourethral glands also known as Cowper's glands are accessory sexual organs that secrete an alkaline mucus-like fluid which neutralizes traces of acidic urine in the urethra and helps with neutralizing the acidity of the vagina and urethral lubrication during ejaculation. They consist of two main glands, which are located ventrally and on either side of bulbar urethra at the level of the urogenital diaphragm. Cowper's glands ducts enter the bulbous urethra near the midline by piercing through the corpus spongiosum [1, 2].

Cystic dilatation of these ducts is uncommon and is called “Cowper's syringocele” [3]. This term has been classified by Maizels et al. into four subtypes: (1) simple syringocele, which is a minimal dilation of the duct, (2) perforate syringocele, which is a duct that resembles a diverticulum and drains into the urethra through a patulous ostium, (3) imperforate syringocele, which is a bulbous duct, which appears like a submucosal cyst, and lastly (4) a ruptured syringocele, which is the remaining fragile membrane after a duct ruptures in the urethra [3].

Traditionally, Cowper's syringocele has been known as a condition affecting pediatric population; however, it is rarely diagnosed in adults [4]. These cysts can be asymptomatic or cause lower urinary tract symptoms by compressing the urethra. Herein we report a case of a 29-year-old male with an imperforate Cowper's gland syringocele.

## 2. Case Report

**Figure 1:**
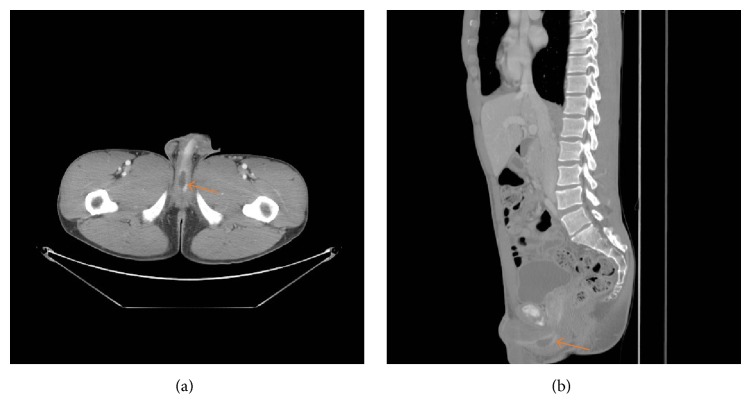
Representative (a) axial and (b) sagittal computed tomography images of the pelvis with contrast demonstrating Cowper's syringocele (arrows).

**Figure 2:**
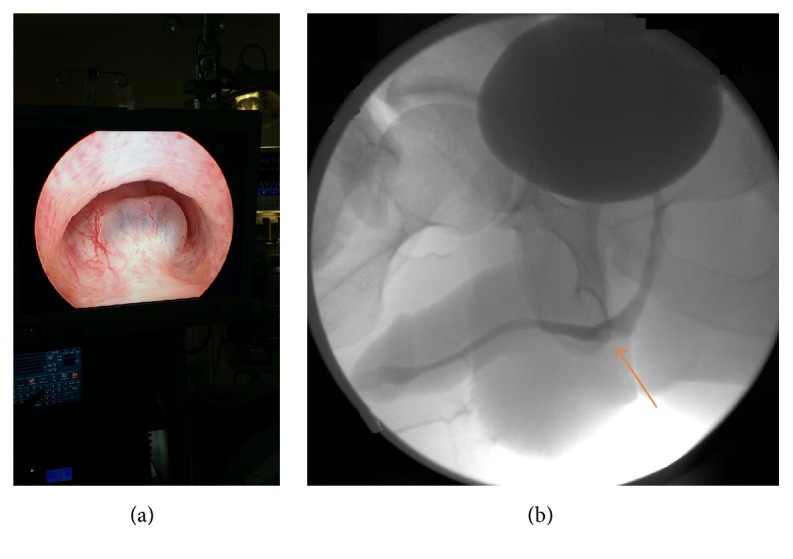
(a) Cystoscopy showing a cystic bulge in the bulbar urethra consistent with syringocele. (b) Retrograde urethrogram showing a filling defect in the bulbar urethra (arrow) consistent with syringocele.

**Figure 3:**
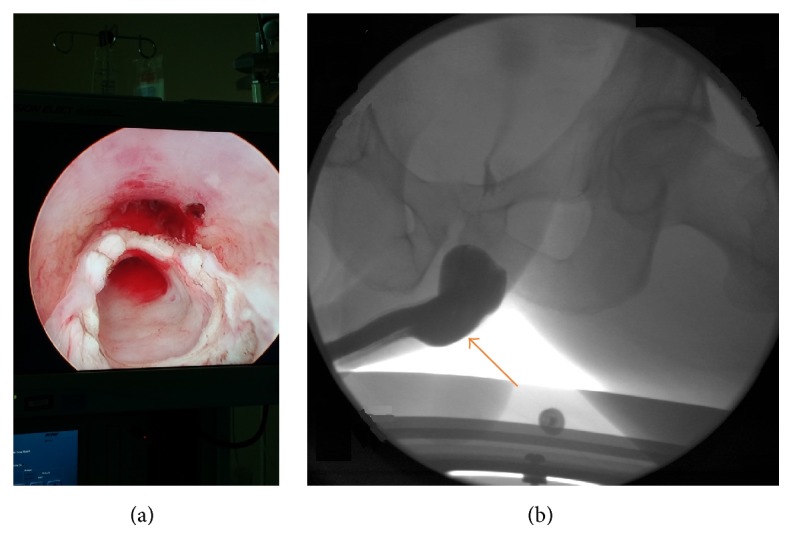
(a) Urethroscopy following transurethral unroofing between the syringocele and the bulbar urethra. (b) Retrograde urethrogram showing a widened bulbar urethra following unroofing of syringocele (arrow).

A 29-year-old healthy man presented to the emergency room with progressive history of urinary frequency, urgency, weak stream, and sense of incomplete emptying over the previous year. His postvoid residual was 300 mL. Urine analysis showed > 3 RBC/Hpf, which prompted a computed tomography (CT) with and without contrast and it showed a cystic lesion around the bulbar urethra (Figure 1). Cystoscopy and retrograde urethrography showed a cyst and filling defect, respectively, bulging into the bulbar urethra without communication into the urethra (Figure 2). A urethral catheter was placed for a few days, and he underwent percutaneous cyst aspiration by interventional radiology through the perineum. He experienced a temporary improvement but had worsening obstructive symptoms over the ensuing 8 months. Ultrasound and cystoscopy confirmed the recurrence of the imperforate Cowper's syringocele. Subsequently a transurethral unroofing of the syringocele using loop cautery was performed, creating a wide communication between the urethra and the syringocele (Figure 3). A urethral catheter was maintained for 1 week and the patient completed a 7-day course of ciprofloxacin. He was asymptomatic at 1-year follow-up. There was no retrograde ejaculation, and his erectile dysfunction rates were unaffected. Repeat urethroscopy demonstrated persistence of the communication between the urethra and syringocele.

## 3. Discussion

The genesis of Cowper's syringocele is not well understood. It has been hypothesized that it could result from a congenital retention cyst of Cowper's gland main duct; this could be particularly true when present in pediatric patients [5]. However, it has also been proposed that it could be acquired in adults. In a case series study by Bevers et al., 6 out of 7 syringocele cases in the series had previous histories of urinary tract infections (UTIs) or trauma [6].

Maizels et al.'s [3] classification of syringoceles lacks clinical significance. Studies have proposed reclassifying them according to the duct's orifice configuration with the urethra and their symptomology into 2 groups: (1) closed or obstructive; (2) open or nonobstructive [6–8]. Closed syringoceles can present with obstructive voiding symptoms, dysuria, perineal pain, and/or urinary retention such as in our case, while open syringoceles may present with postvoid dribbling, urethral discharge, recurrent UTIs, perineal pain, and/or hematuria [6–8].

The true prevalence of Cowper's syringocele is not known; however, 32 adult cases have been reported in the literature as of 2012 [9]. Bevers et al. report a case series of 7 cases diagnosed within 18 months [6]. Therefore, it can be speculated that some cases may go undiagnosed or misdiagnosed. Physicians should have an index of suspicion in young patients with lower urinary tract symptoms or recurrent UTIs in male patients. Syringoceles were visualized by ultrasound (US) at the anatomic region of Cowper's gland in some cases [4, 10, 11]; however, whenever US results are questionable, retrograde urethrography should be done, which confirms the diagnosis [12]. Other modalities such as cystourethroscopy, CT, and magnetic resonance imaging (MRI) can confirm the diagnosis as well [8].

Asymptomatic syringoceles have been observed [13]. In Bevers et al. case series, 3 cases resolved on their own with a maximum follow-up interval of 18 months [6]. In symptomatic cases, a minimally invasive procedure is usually successful. With a maximum follow-up interval of 23 months (mean 12 months), 4 patients were symptom-free after they underwent unroofing or marsupialization of the cyst by a Collins knife [6]. Unroofing with the holmium (YAG laser) was successful in another case report as well [14]. We performed unroofing with loop cautery, and the patient remained asymptomatic at 1-year follow-up. We believe loop cautery provides the theoretical advantage of wider communication between the cyst and the urethra and therefore less chance for recurrence. Open procedures such as transperineal ligation and open excision are usually performed after failed transurethral unroofing [15, 16].

## 4. Conclusion

Physicians should have an index of suspicion when young male adults present with lower urinary tract symptoms. Transurethral unroofing or marsupialization with loop cautery by opening the syringocele was successful in our symptomatic patient.
